# The Reclassification of a *FBN1* Variant of Unknown Significance Associated With Marfan Syndrome Through Careful Clinical Correlation and Family-Based Evaluation

**DOI:** 10.1155/carm/8854360

**Published:** 2025-06-02

**Authors:** Dominique Bouhamdani, Véronique Allain, Nadia Bouhamdani, Mouna Ben Amor

**Affiliations:** ^1^Research and Health Evaluation Department, Vitalité Health Network, Moncton, New Brunswick, Canada; ^2^New Brunswick Center for Medical Education, University of Sherbrooke, Moncton, New Brunswick, Canada; ^3^Medical Genetics Department, Vitalité Health Network, Moncton, New Brunswick, Canada

**Keywords:** case report, *FBN1*, Marfan syndrome, ocular abnormalities

## Abstract

**Background:**Fibrillin-1 (FBN1) is a major structural component of the extracellular matrix, providing strength and stability to tissues. Pathogenic variants lead to the development of *FBN1*-associated syndromes which comprise a broad host of phenotypes, and more commonly, Marfan syndrome (MFS). MFS is typically diagnosed in patients presenting with ectopia lentis, thoracic or aortic disease, and skeletal features, which may prompt genetic testing.

**Case Presentation:** In this case report, we describe the reclassification of a newly identified heterozygous *FBN1* variant, c.2686T > A, p.(Cys896Ser), to likely pathogenic in a Caucasian 21-year-old female patient presenting with abnormal anterior eye segment with superior bilateral ectopia lentis; joint pain affecting wrists, knees, and upper back; and mild thoracolumbar scoliosis. Identification of this variant led to cascade testing in the patient's 49-year-old mother which revealed the same *FBN1* variant and an incidental finding of aortic dilatation, prompting standard management. Notably, the identification and reclassification of the variant led to early diagnosis and preventive management in the patient's mother, including cardiovascular monitoring and treatment. The segregation of the phenotype in both patient and mother, the family member testing, the variant's absence in control populations, and all in silico tools predicting pathogenicity led to the reclassification of this *FBN1* variant to likely pathogenic.

**Conclusion:** We highlight the reclassification of a variant of unknown significance through careful clinical correlation and family-based evaluation. This reclassification led to a timely diagnosis and preventive management through cascade testing, demonstrating the real-world utility of genetic testing and cascade screening in connective tissue disorders.

## 1. Background

Fibrillin-1 (*FBN1*), located on chromosome 15q21.1, is a major structural component of the extracellular matrix, namely, microfibrils, which provide strength and stability to tissues that are under constant stretch and recoil [1]. In addition, microfibrils have emerged as being able to sequester transforming growth factor (TGF)-β superfamily of growth factors [2], diversifying their biological function. *FBN1*-associated syndromes comprise a broad range of phenotypes, the most common being Marfan syndrome (MFS).

MFS is a dominantly inherited disorder affecting connective tissue with multisystem involvement [1]. Most *FBN1* variants are inherited from a parent and roughly 25% arise de novo; substantial intrafamilial and interfamilial variability is observed in terms of disease manifestation [3]. Classically, ocular, cardiovascular, and musculoskeletal abnormalities are observed, and to a lesser extent, involvement of the lung, skin, and central nervous system [3]. More specifically, dislocation of the lens, cataract, myopia, and retinal detachment occur in the eyes, and cardiovascular system involvement most commonly includes aortic dilatation, regurgitation, and aneurysms [4]. Notably, cardiac complications such as aortic dilatation, dissection, and rupture, in addition to involvement of the aortic and mitral valves reduce life expectancy considerably [5]. Skeletal deformities include thoracolumbar scoliosis, dolichostenomelia, arachnodactyly, pectus deformities, and joint hypermobility, as well as progressive deformities such as protrusion acetabulae and dural ectasia [5]. Other *FBN1*-associated diseases include, but are not limited to, Weill–Marchesani syndrome, acromicric dysplasia, isolated familial ectopia lentis, and skeletal features of MFS without ectopia lentis and aortic disease [1]. Regardless, all patients with a pathogenic *FBN1* variant are at risk of developing cardiovascular, skeletal, and ophthalmologic complications [6]. Typically, MFS is diagnosed in patients presenting with classic ectopia lentis, thoracic or aortic disease, and mild skeletal features, which may prompt genetic testing in the proband and ensue cascade testing in family members [7, 8]. Furthermore, given the similarities with other hereditary diseases such as Loeys–Dietz, multigene panels can greatly help in the differential diagnosis, thus impacting patient care and management [1].

Herein, we describe a newly identified heterozygous *FBN1* initially reported as a variant of unknown significance (VUS), c.2686T > A, p.(Cys896Ser), in a patient with abnormal anterior eye segment with superior bilateral ectopia lentis and skeletal features. Identification of this variant led to cascade testing in the patient's mother which revealed the same *FBN1* variant. Referral to a cardiologist led to an incidental finding of aortic dilatation, prompting standard treatment and management as MFS. This clinical report illustrates the importance of genetic testing and how to interpret and act upon VUS findings based on a family study and variant segregation. Furthermore, it illustrates the need for comprehensive familial genetic evaluations, genotype/phenotype correlation, VUS reclassification, and the possibility of cascade screening as well as preventive measurement in at risk family members.

## 2. Methods

### 2.1. Case Presentation

A Caucasian 21-year-old female, the youngest child of nonconsanguineous Canadian parents, was evaluated at the New Brunswick medical genetics clinic upon referral from an ophthalmologist regarding ocular anomalies in the anterior eye segment, more specifically, bilateral superior ectopia lentis for which the patient was treated with lensectomy. The ophthalmology consult was prompted because of a significant drop of vision associated with photophobia and blurred vision without diplopia or bright spots in the field of vision. A history of strong myopia since the age of 6 was of noted. The patient also presented mild skeletal features, more specifically, vague joint pain affecting upper back, wrists, knees, and the left ankle, which had been fractured in childhood, as well as mild thoracolumbar scoliosis. A history of luxation or subluxation of joints and hyperlaxity were absent. The patient did not have a history of abdominal hernias or genitourinary or rectal prolapse, no coagulation disorders nor abnormal skin scarring, and no thromboembolic event.

Upon physical examination, the patient's height was at the 75th percentile. The physical exam showed a decrease in the upper to lower body ratio, and normal arm span to height ratio; the patient had thin lips, mild prominence of the columella, a normal palate, and non–bifid uvula. The musculoskeletal exam was only relevant for mild thoracolumbar scoliosis, and the patient did not have pectus, flat feet, or joint hyperlaxity, and the wrist and thumb signs were both negative. Cardiopulmonary, abdominal, neurological, and tegument exams were also normal. Microphthalmia, anophthalmia, and eye anterior segment dysgenesis multigene panel were requested to further complement the investigation; the differential diagnosis included MFS and other related connective tissue disorder; nonsyndromic familial ectopia lentis associated with some mutations such as *FBN1*, *ASPH*, and *ADAMTSL4*; and homocystinuria. Genetic testing identified a heterozygous VUS, namely, FBN1 (c.2686T > A, p.(Cys896Ser)).

The family history was positive for congenital aphakia in the mother with no other clinical findings suggestive of a connective tissue disorder and no other relatives with obvious features of MFS or a connective tissue disorder. However, cascade testing found that the patient's mother also presented the same *FBN1* variant, supporting a hereditary mode of inheritance. This discovery prompted a cardiovascular evaluation in the mother and heart ultrasound which identified a mild aortic dilatation at 13 mm with a Z-score at 2.24. Based on ectopia lentis and the aortic dilatation, MFS diagnosis was made in the mother by the cardiologist. A preventative bisoprolol treatment (5 mg, once daily) was prescribed as well as yearly baseline ultrasound follow-ups. Despite the lack of cardiovascular manifestation in the daughter on exam and on heart ultrasound at the time of initial evaluation, the described eye phenotype, the musculoskeletal abnormalities, and the diagnosis in the mother (carrying the same *FBN1* variant), MFS was strongly suspected, and yearly motoring of the heart was also prescribed. A medical genetic evaluation was consequently prompted in two unaffected sisters who had no musculoskeletal or ocular findings. They were both noncarriers of the maternal *FBN1* variant.

### 2.2. Exome Library Construction, Sequencing, and Analysis

Blood samples were collected from the patient and their mother with written informed consent. DNA extraction, sequencing, and data analysis were performed by BLUEPRINT GENETICS (BLUEPRINT GENETICS OY, Keilaranta 16 A-B, 02150 Espoo, Finland) and are briefly described as follows. The total gDNA was extracted and fragmented by isothermal sonochemistry processing, and regions of interest were then targeted using the hybridization-based target capture method. The prepared sequencing libraries were then sequenced using the Illumina platform's sequencing-by-synthesis method using paired-end sequencing. The primary data analysis was done with Illumina's proprietary software, and the identified variants were classified according to the Blueprint Genetics Variant Classification Schemes modified from the 2015 ACMG guidelines.

## 3. Results

### 3.1. Multigene Panel Analysis

The sequencing data identified a heterozygous *FBN1* variant (c.2686T > A, p.(Cys896Ser)) classified as uncertain significance (absent from the gnomAD database). The variant was predicted to be deleterious by in silico tools; parameters included POLYPHEN which described the variant as probably damaging, SIFT which described the variant as deleterious, and Mutation Taster which described the mutation as disease-causing. The affected amino acid was determined to be highly conserved across mammals, which indicated that the position may not tolerate variation. Hence, based on the family genetic evaluation, the heart ultrasound findings in the mother and molecular results, the molecular laboratory reclassified the identified *FBN1* variant of uncertain significance as a likely pathogenic.

The lack of functional validation of variant remains a limiting factor; however, the combination of clinical findings, family segregation data, in silico predictions, and codon-level evidence supports the likely pathogenic classification. Additional limitations include the classification scheme which deviates slightly from ACMG criteria. Thus, we cannot provide strict ACMG classification with adjustments to strength. However, pathogenicity is based on the following: (i) clear family gene-phenotype association, (ii) absence in control populations (comparable to PM2), (iii) predicted deleterious by all in silico tools (comparable to PP3), (iv) well-characterized variants at the same codon are deleterious (could be comparable to PM5), and (v) the domain affected is a key functional domain (could be comparable to PM1).

## 4. Discussion

We report a patient presenting ocular anomalies, more specifically, severe myopia with bilateral superior ectopia lentis, in association with a heterozygous *FBN1* variant (c.2686T > A, p.(Cys896Ser)), which was previously classified as a VUS and not yet documented in the literature. The variant identified arises in the TGFβ-binding protein-like (TB) domain of FBN1 which is characterized by cysteine residues that form disulphide bonds [9]. Disturbance in the highly conserved disulfide bonds cause misfolding of the protein and disrupt protein-protein interaction [9], thus preventing assembly and deposition of microfibrils into the extracellular matrix [9]. More specifically, the variant identified is in the fourth TB domain, which has been functionally linked to integrin binding with suspected links to MFS [10]. Notably, *FBN1* variants that disrupt cysteine residues are more common in patients with MFS presenting with ectopia lentis [11, 12]. They are also more strongly associated with patients that have cardiovascular manifestation, mostly aortic dilatation and dissection, caused by alterations in the composition of the medial layer of the aorta, but also mitral and tricuspid valve prolapse and regurgitation [13, 14]. In addition, three other pathogenic missense variants in the same codon have been reported in MFS with ectopia lentis, namely, c.2688T > G p.(Cys896Trp), c.2686T > G p.(Cys896Gly), and c.2687G > A p.(Cys896Tyr) [13, 15–18], altogether highlighting its functional importance and the pathogenicity of the newly identified variant. However, further molecular insight into the functionality of the variant is needed to confirm this at the cellular level.

Although the proband did present with ocular and musculoskeletal manifestations commonly associated with MFS, namely, significant myopia since childhood, bilateral ectopia lentis, and thoracolumbar scoliosis, no other typical manifestations of MFS was observed (i.e., pectus excavatum or carinatum, aortic dilatation or dissection, skin striae, facial features as retrognathia, dural ectasia, and pneumothorax), and therefore did not meet the Ghent nosology diagnostic criteria [7]. The patient also did not present with typical clinical features of related connective tissue disorders to MFS, such as Ehlers–Danlos syndrome, Beals syndrome, Loeys–Dietz syndrome, or Shprintzen–Goldberg syndrome [19]. The patient did not present any joint hyperlaxity, tissue fragility, contractures, abnormal hand and feet characteristics, skin hyperextensibility, easy bleeding, dural ectasia, or facial abnormalities, such as cleft palate or craniosynostosis [19]. Although the patient presented with strong myopia, bilateral ectopia lentis, and scoliosis, which is associated with homocystinuria, the patient did not have other typical characteristics of homocystinuria such as developmental delay, arachnodactyly, dolichostenomelia, movement disorders, or pectus excavatum [19]. However, cascade testing found that the patient's mother, known for congenital aphakia, also presented the same *FBN1* variant, prompting a cardiovascular evaluation which revealed mild aortic dilatation. Based on ectopia lentis and the aortic dilatation, MFS diagnosis was made in the mother. Despite the lack of cardiovascular manifestation in the daughter at the initial evaluation, MFS diagnosis is very likely, and yearly motoring of the heart is planned.

If a timely diagnosis had not been made based on the lack of Ghent nosology diagnostic criteria for MFS, asymptomatic or undiagnosed aortic root aneurysms may have progressed into fatal acute aortic dissection/rupture. Notably, patient outcomes and prognosis are dramatically improved with an early diagnosis, which aim to delay or prevent the progression of aortic dilatation and fatal complication [1]. It is important to identify potentially pathogenic *FBN1* variant, as there is significant probability that patients will present with incomplete phenotype at initial assessment and might develop complications later in life [3]. These patients must be followed up regularly, mainly with ocular, cardiovascular, and musculoskeletal exams before complications arise [3, 20]. They also need adequate treatment according to their clinical presentation, given high mortality rates of untreated patients [3]. There is also significant impact for the patient's offspring, as the variants follow autosomal dominant inheritance [3]. Hence, as illustrated in this clinical report, even in the absence of apparent cardiovascular abnormalities, the presence of ocular and musculoskeletal manifestations should prompt cascade testing of family members as it can lead to (i) a reclassification of a genetic VUS, (ii) a timely diagnosis, and consequently, (iii) preventative efforts and tailored care that may delay disease presentation and complications. Altogether, this clinical report illustrates the importance of VUS reclassification based on a thorough family evaluation, clinical correlation, in silico predictions, codon-level evidence and segregation. Notably, this combinatory approach comprised of a comprehensive familial genetic evaluation and molecular results led to a diagnostic confirmation and appropriate management in at risk relatives.

## 5. Conclusion

In conclusion, we describe an undocumented *FBN1* variant (c.2696T > A, p.(Cys896Ser)) which was initially classified as a VUS. Family member testing and the established association between the gene and the patient's phenotype, the variant's absence in control populations, in silico predicted pathogenicity, and previously reported well-characterized variants at the same codon have led to the reclassification of this variant as likely pathogenic. Furthermore, we demonstrate the real-world utility of a comprehensive familial genetic evaluations and cascade screening in connective tissue disorders, which enabled a timely diagnosis and preventive management in the patient's mother.

## Data Availability

The datasets analyzed during the current study, apart from those presented, are not publicly available due to their identifiable nature.

## Nomenclature

FBN1: Fibrillin-1
MFS: Marfan syndrome
TGF: Transforming growth factor
